# Effect of 2000-Hour Ultraviolet Irradiation on Surface Degradation of Glass and Basalt Fiber-Reinforced Laminates

**DOI:** 10.3390/polym17141980

**Published:** 2025-07-18

**Authors:** Irina G. Lukachevskaia, Aisen Kychkin, Anatoly K. Kychkin, Elena D. Vasileva, Aital E. Markov

**Affiliations:** 1 Federal Research Centre “The Yakut Scientific Centre of the Siberian Branch of the Russian Academy of Sciences”, 2 Petrovskogo Str., Yakutsk 677000, Russia; icen.kychkin@mail.ru (A.K.); aital.markov@gmail.com (A.E.M.); 2 V.P. Larionov Institute of Physical and Technical Problems of the North Siberian Branch Russian Academy of Sciences, 1 Oktyabrskaya Str., Yakutsk 677980, Russia; kychkinplasma@mail.ru; 3 Institute of Physics and Technology, North-Eastern Federal University named after M.K. Ammosov, 58 Belinsky Str., Yakutsk 677000, Russia; vasilyeva_edm@mail.ru

**Keywords:** UV chamber, UV irradiation, polymer composite materials, fiber-reinforced laminates, epoxy matrix, photodegradation, surface degradation

## Abstract

This study focuses on the influence of prolonged ultraviolet (UV) irradiation on the mechanical properties and surface microstructure of glass fiber-reinforced plastics (GFRPs) and basalt fiber-reinforced plastics (BFRPs), which are widely used in construction and transport infrastructure. The relevance of the research lies in the need to improve the reliability of composite materials under extended exposure to harsh climatic conditions. Experimental tests were conducted in a laboratory UV chamber over 2000 h, simulating accelerated weathering. Mechanical properties were evaluated using three-point bending, while surface conditions were assessed via profilometry and microscopy. It was shown that GFRPs exhibit a significant reduction in flexural strength—down to 59–64% of their original value—accompanied by increased surface roughness and microdefect depth. The degradation mechanism of GFRPs is attributed to the photochemical breakdown of the polymer matrix, involving free radical generation, bond scission, and oxidative processes. To verify these mechanisms, FTIR spectroscopy was employed, which enabled the identification of structural changes in the polymer phase and the detection of mass loss associated with matrix decomposition. In contrast, BFRP retained up to 95% of their initial strength, demonstrating high resistance to UV-induced aging. This is attributed to the shielding effect of basalt fibers and their ability to retain moisture in microcavities, which slows the progress of photo-destructive processes. Comparison with results from natural exposure tests under extreme climatic conditions (Yakutsk) confirmed the reliability of the accelerated aging model used in the laboratory.

## 1. Introduction

The choice of reinforcing filler and polymer matrix plays a key role in determining the performance characteristics of fiber-reinforced polymer composites (FRPs) intended for use in harsh climatic conditions. According to the results of a comparative analysis [1], basalt fiber (BFRP) exhibits higher values of elastic modulus (~85–90 GPa), tensile strength (~3.0 GPa), and thermal resistance (up to 400 °C) compared to glass fiber (GFRP: ~70–76 GPa, ~2.5–3.0 GPa, up to 300 °C, respectively), while maintaining comparable cost. Compared to carbon fiber composites (CFRP: >200 GPa modulus, >3.5–5.5 GPa tensile strength, >500 °C thermal resistance), BFRP are inferior in mechanical performance but offer advantages in terms of environmental safety due to their natural origin and the absence of additional chemical processing.

The durability of polymer composites under environmental exposure is largely determined by the properties of the polymer matrix, including its resistance to hydrolysis, photodegradation, and thermo-oxidative degradation. The strength of the interfacial bonding between the reinforcing fiber and the matrix also plays a critical role, as confirmed by recent research findings [2,3].

In the context of matrix systems, epoxy resins remain the preferred choice due to their high adhesion, dimensional stability, and resistance to climatic factors, despite their non-recyclability [4,5]. They are widely used in coatings [6,7], adhesives [8], and in composites reinforced with glass and basalt fibers. The combination of these properties makes BFRP–epoxy composites highly promising for infrastructure and construction applications, especially where prolonged exposure to sunlight and moisture is expected.

However, the durability of such materials is significantly limited by climatic factors, particularly UV radiation, which initiates photochemical reactions leading to degradation of the polymer matrix and, consequently, to a decline in mechanical properties [9,10]. The photodegradation mechanism includes chain scission and oxidative processes activated by radiation in the 295–400 nm wavelength range [11,12].

An additional adverse factor is increased moisture uptake, especially under conditions of prolonged humidity and elevated temperatures. UV degradation promotes moisture ingress, pore formation, and microcracking in the matrix, leading to delamination and weakened fiber–matrix adhesion [7,13]. Water primarily penetrates through the matrix, triggering physicochemical processes that may reduce interlaminar shear strength (ILSS) by 10–50%. Starkova et al. [14] showed that for CFRP composites subjected to moisture at 80 °C, residual strength may decrease to 72.8% of the original value.

The synergistic effect of temperature and moisture significantly accelerates the degradation of the mechanical properties of polymer composites. Elevated temperatures enhance the diffusion of water into the polymer matrix and along interfacial boundaries, leading to the accelerated plasticization of the resin, the formation of microcracks, and a reduction in interfacial strength. According to the comprehensive findings presented by Li et al. [15], the combined influence of temperature, hydrostatic pressure, and salinity significantly increases water absorption, which is accompanied by a noticeable decline in the mechanical performance of hybrid reinforcement rods made of carbon and glass fibers.

Another important aspect is the size effect, as demonstrated in a recent study [16], which found that the degree of strength degradation in BFRP under climatic aging increases with decreasing sample diameter due to the dominance of surface damage. This underscores the need to consider the combined effects of moisture absorption, temperature, and geometry in the design of durable FRP structures.

Several previous studies have confirmed that UV exposure significantly affects the properties of polymer composites. Lu et al. [17] developed a UV degradation model incorporating damage kinetics and environmental parameters. Shi et al. [18] demonstrated failure mechanisms in CFRP, including surface cracking and strength loss. Wu et al. [19] and Khotbehsara et al. [20] investigated chemical degradation and visual signs of aging in epoxy systems, such as yellowing and surface cracking. Other works, including those by Mohammad [21] and Kazi et al. [22], reported reduced mechanical properties of GFRPs and BFRPs under UV exposure.

While these studies provide valuable insights into UV aging effects, most lack a systematic assessment of the full degradation process. Typically, authors focus on isolated aspects—strength loss, discoloration, surface erosion, or theoretical modeling. However, the coupled analysis of mechanical, geometrical, and microstructural changes, along with validation of laboratory models under real climatic conditions, remains underexplored.

To address these gaps, the present study implements an integrated experimental approach. UV aging was conducted in a custom-built laboratory chamber, with the simultaneous monitoring of flexural strength, surface roughness, mass, thickness, and depth of degraded layers. Special attention was given to the moisture-retaining effect of surface micro-roughness as a potential stabilizing factor. Laboratory results were compared with long-term natural weathering data from the extreme climatic region of Yakutsk, providing new insights into UV degradation mechanisms and enabling more accurate durability predictions for FRP materials.

## 2. Materials and Methods

To assess the effects of UV radiation on glass and basalt fiber-reinforced plastics, the specimens were divided into groups based on exposure time: control samples (0 h) that were not subjected to radiation and served as a reference; samples after 500 h of irradiation, which revealed initial changes in structure and properties; samples after 1000 h, representing intermediate stages of degradation; samples after 1500 h, demonstrating the development of accumulated effects from prolonged exposure; and samples after 2000 h, showing the final stages of photodegradation of the polymer matrix and fiber reinforcement. This distribution enables the comprehensive evaluation of the dynamics of structural and mechanical changes at various stages of aging under UV exposure.

The binder system consisted of CYD128 epoxy resin (Hefei TNJ Chemical Industry Co., Ltd., Hefei, China) with a mass fraction of 56.7%, cured with iso-methyltetrahydrophthalic anhydride (iso-MTHPA) produced by JSC “Sterlitamak Petrochemical Plant” (Sterlitamak, Republic of Bashkortostan, Russia) at 42.5 wt.% and using 2,4,6-tris(dimethylaminomethyl)phenol (accelerator UP606/2, also from JSC “Sterlitamak Petrochemical Plant”) at 0.8 wt.%. The binder formulation was prepared in accordance with the technical specification RTP-SP220994511-1999T.

As reinforcing fillers, the following fabrics were used:

For GFRP: Glass fabric TR-560-30A (100), manufactured by JSC “Polotsk-Steklovolokno” (Polotsk, Belarus).

For BFRP: Basalt fabric BT-11 (100), manufactured by LLC “Technical Fabric Factory” (Vladimir, Russia).

The technical properties of the constituent materials are presented in Table 1.

**Figure 1:**
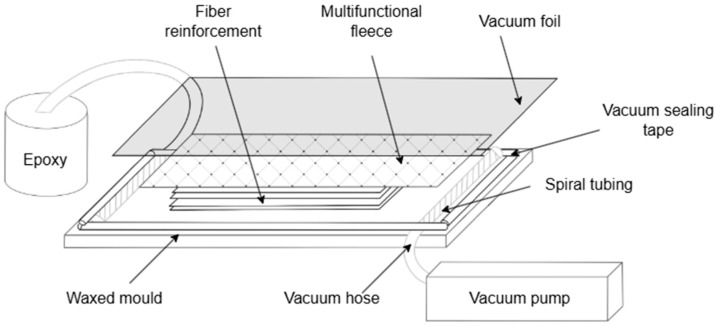
Schematic representation of vacuum-assisted resin infusion process for fabrication of glass and basalt fiber-reinforced composite specimens.

Composite plates with dimensions 950 × 450 × 5 mm were manufactured using the vacuum infusion method, followed by curing in molds at a temperature of 160 ± 2 °C for 4 h. The composition and layer configuration of the plates are shown in Table 2. The process setup is illustrated in Figure 1. The thickness of the samples was measured using an MK-25 micrometer with a division value of ±0.001 mm.

Surface inspection was carried out using a HighCloud optical microscope at 20× magnification, allowing for the detection of microcracks, defects, and structural changes in the polymer matrix.

Sample thickness was measured using an MK-25 micrometer with a resolution of ±0.001 mm in accordance with GOST 33846–2016, while the mass before and after UV exposure was determined using VL-320C electronic scales with a precision of ±0.001 g.

Fourier-transform infrared (FTIR) spectroscopy was performed using a Varian 7000 FT-IR spectrometer (Agilent Technologies, Santa Clara, CA, USA). Spectra were recorded in the range of 4000–400 cm^−1^ with a resolution of 4 cm^−1^, averaging 32 scans per sample. Prior to measurement, all specimens were conditioned under dry ambient conditions. The analysis was conducted in ATR (attenuated total reflectance) mode, providing high reproducibility and accuracy in detecting surface chemical changes in the composites after UV exposure.

To quantitatively assess surface topography, a Mitutoyo SJ-201 contact profilometer was used to record roughness parameters and microdefect depth. Each measurement was performed at three different surface points, and the values were averaged.

Mechanical properties were evaluated by three-point bending tests following GOST R 56810–2015. The tests were conducted on a Zwick Roel Z600 universal testing machine (model BRS-F0600TN.R09, serial number: 160088–2008, manufactured by Zwick, Ulm, Germany) at the Shared Research Facility of the Federal Research Center “Yakutsk Scientific Center of the Siberian Branch of the Russian Academy of Sciences”.

At each time interval, no fewer than five specimens of each type (GFRP and BFRP) were tested.

## 3. Test Equipment

**Figure 2:**
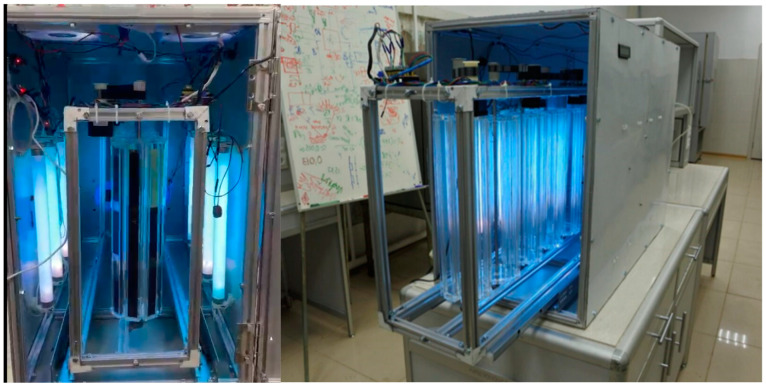
External view of developed UV camera.

To study the effects of non-uniform aging of polymer composite materials (PCMs) under ultraviolet (UV) exposure, a custom-built UV chamber (Figure 2) was developed. The chamber has dimensions of 84 × 41 × 64 cm, with a distance of 11 cm between the lamps and the sample holders. The laboratory UV chamber is equipped with eight fluorescent lamps: four LE 10 N (UVB, peak emission ~313 nm) and four LUFT 10 (UVA, peak ~365 nm). The use of two lamp types was intended to simulate the full solar UV spectrum (295–400 nm), encompassing both short- and long-wave UV radiation that affects polymers outdoors. This configuration is in accordance with the ASTM G154 standard [23] for accelerated UV aging using UVA-340 and UVB lamps.

The total irradiance within the chamber is 2.68 W, corresponding to a UV intensity of 7.88 W/m^2^ on the sample surface. Up to 120 specimens can be simultaneously irradiated, with uniform exposure ensured over an area of 0.34 m^2^, depending on the holder configuration. This design allows for spectrally and energetically representative simulation of solar exposure under controlled conditions, making the laboratory model suitable for comparative durability assessments.

The sample holders are mounted on a stepper mechanism that enables even the exposure of all sample sides. Cooling elements are integrated into the chamber housing and connected to a microcontroller to prevent local overheating near the UV sources. The temperature and humidity were not actively controlled and remained at ambient laboratory conditions (approximately 20–25 °C and 45–55% relative humidity), reflecting a passive environmental approach.

To investigate degradation gradients and their effects on mechanical properties, the chamber was equipped with holders for bending test specimens of 11 × 2 × 0.5 cm size, providing an irradiated surface area of ~22 cm^2^. The three-point bending test is particularly informative as it accounts for normal and shear stress distribution across the sample thickness and differentiates the tensile and compressive behavior of composite layers under asymmetric degradation conditions.

Non-uniform UV exposure was achieved by irradiating only one side of the sample, while the opposite side was fully shielded by the fixture, thus avoiding UV contact. This setup created a controlled through-thickness degradation gradient, simulating the asymmetric aging typical of outdoor service conditions, where UV exposure is limited to one surface.

Fluorescent lamps were selected due to their advantages over other UV sources [17,18,19,24], including reliability, stable emission spectrum throughout service life, and low energy consumption at high UV intensity. The chamber design facilitates simultaneous irradiation of a large number of samples, while maintaining a lamp-to-sample distance of at least 10 cm. This layout ensures the efficient use of UV output, minimizes energy losses, and avoids local overheating.

To evaluate the influence of UV dose, samples were irradiated for 2000 h, simulating several years of natural sunlight exposure. The main distinction from natural weathering lies in the stability of irradiation intensity and the absence of variable environmental factors. In contrast to the real outdoor environment—characterized by fluctuations in irradiation, precipitation, contamination, and temperature—the laboratory chamber ensures standardized, accelerated the aging free of confounding variables. To validate the model, the laboratory results were compared with long-term natural exposure data from the extreme climate of Yakutsk.

## 4. Results and Discussion

Changes in Appearance and Surface Degradation of the Samples

It is well established that solar radiation, particularly its ultraviolet (UV) component, is one of the critical factors influencing the performance of polymer composite materials (PCMs) [25,26,27,28]. Exposure to solar radiation induces the photodegradation of the material’s surface, leading to stress accumulation, crack formation, and the deterioration of the matrix surface. This, in turn, results in exposure of the reinforcing filler layers, thereby reducing the load-bearing capacity of the material. As UV-induced surface erosion and degradation progress, the thickness of the sample may decrease, while the number of surface defects and the amount of absorbed moisture increase—ultimately contributing to a reduction in mechanical strength.

**Figure 3:**
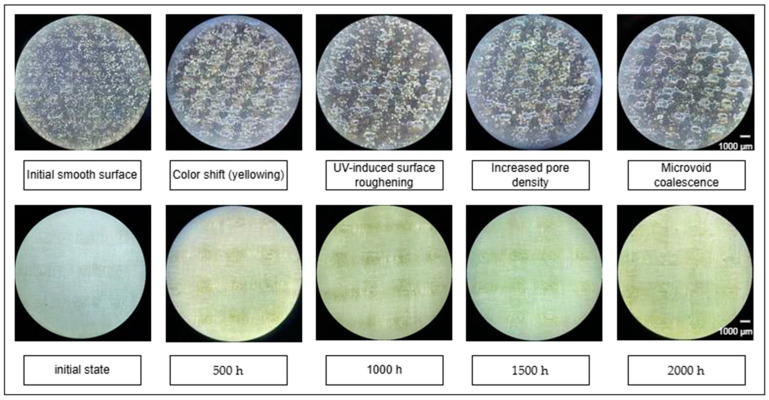
Appearance of samples after UV exposure: top row—basalt fiber-reinforced plastic (BFRP); bottom row—glass fiber-reinforced plastic (GFRP).

The inspection and visual examination of the specimen surfaces during UV chamber exposure—conducted at intervals of 0, 500, 1000, 1500, and 2000 h—are presented in Figure 3.

The initial inspection of glass fiber-reinforced plastic (GFRP) specimens revealed a light, homogeneous surface free from defects. However, as early as 500 h, visible yellowing and the appearance of small surface pores were observed, likely associated with the onset of thermo-photo-oxidation of the epoxy matrix. After 1000 h, the surface became less uniform, with noticeable discoloration. By 1500 h, these changes were more pronounced: the surface developed a yellowish hue, and defect formation intensified, indicating progressive photo-oxidative degradation of the resin.

The visual inspection of basalt fiber-reinforced plastic (BFRP) samples showed no significant initial surface changes. However, after 500 h of UV exposure, the enlargement of pre-existing pores in the surface layer became apparent. With continued exposure—particularly after 2000 h—these defects deepened and widened, indicating the advancement of micro-destructive processes. These changes were clearly documented through profilometric analysis, providing additional evidence of UV-induced surface degradation.

The thickness of the specimens was measured using an MK-25 micrometer with a resolution of ±0.001 mm, in accordance with GOST 33846-2016 [29]. The thickness (*d*) of the FRPs was recorded at 20 uniformly distributed points across the surface of each specimen. The values present in Table 3 represent mean values calculated from a series of at least five specimens, while the values following the “±” sign indicate the standard deviation (SD).

**Figure 4:**
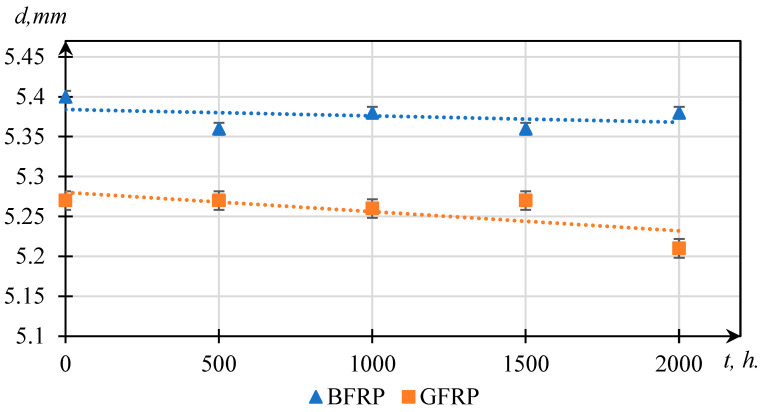
Thickness variation of laminated composites after UV exposure for 0, 500, 1000, 1500, and 2000 h.

Figure 4 presents the changes in composite thickness as a function of UV exposure time. The graph indicates a gradual decrease in thickness for both GFRPs and BFRPs, with more pronounced variations in the GFRP samples, particularly after 1000 h. Although these dimensional changes are relatively small and near the measurement accuracy limit, they may reflect early signs of structural alteration in the material. The results suggest that GFRPs are more susceptible to UV-induced degradation processes compared to BFRPs, which shows a more stable dimensional response under similar conditions. However, these observations are interpreted with caution and are further supported by FTIR spectroscopy data.

The analysis of thickness changes in composite materials under UV exposure revealed distinct differences in the degradation behavior of GFRPs and BFRPs. In BFRP specimens, an increase in thickness was observed after 1500 h of irradiation, likely due to the development of internal defects—such as interlaminar delamination, blistering, or swelling—resulting from the photochemical degradation of the matrix and the accumulation of residual stresses. In contrast, similar effects were detected in GFRPs already after 1000 h of UV exposure, indicating an earlier and more intense disruption of internal structural integrity. These thickness changes are presumably associated with the breakdown of interfacial bonds, the formation of microvoids, and a decrease in adhesion between the polymer matrix and reinforcing fibers. Such transformations reflect differences in the stability of the interphase layer and the materials’ ability to mitigate internal stresses under photodegradation conditions.

All composite materials were weighed using VL-320C electronic scales (LLC “NPP Gosmetr”) with an accuracy of 0.0001 g and a hydrostatic weighing kit. Mass measurements were taken both before and after UV exposure.

**Figure 5:**
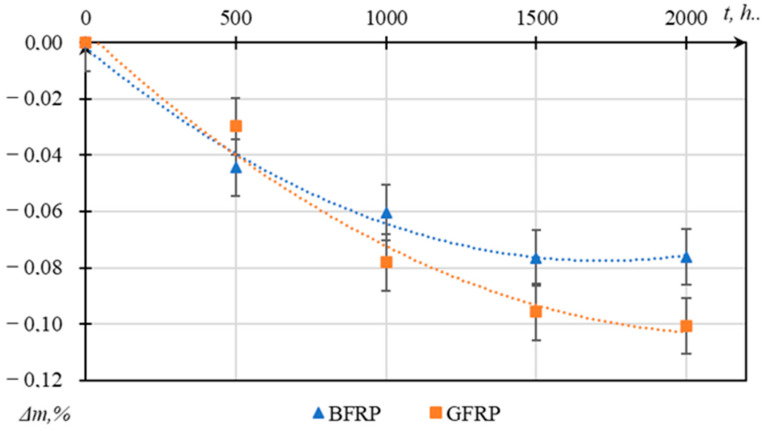
Graph of relative mass changes of GFRP and BFRP samples over UV exposure time.

Analysis of the mass change data presented in Figure 5 indicates that both tested materials exhibit minor but statistically significant mass loss (up to 0.1%) under UV exposure for 2000 h.

BFRPs demonstrated greater stability compared to GFRPs: the maximum mass loss for BFRPs was 0.076%, while for GFRPs it was 0.101%.

The most intense mass loss was observed during the initial exposure period (0–500 h). After 1000 h, the rate of mass loss significantly decreased, indicating the stabilization of degradation processes. Up to 1000 h, GFRPs lost mass approximately 29% more rapidly than BFRPs, underscoring the higher susceptibility of glass fiber composites to UV radiation.

Despite the relatively small absolute values of mass loss (no more than 0.1%), this parameter serves as a sensitive indicator of initial surface degradation of the polymer matrix under UV exposure. The changes were found to be statistically significant (*p* < 0.05, Student’s *t*-test) based on repeated measurements across a sample set of five specimens. The recorded mass loss is attributed to bond scission, the leaching of low-molecular-weight fragments, and the onset of surface erosion.

A comparative analysis of mass change dynamics enables the identification of differences in material resistance during the early stages of aging, before notable mechanical deterioration occurs. In particular, the lower mass loss in BFRPs compared to GFRPs indicates its higher resistance to UV-induced degradation.

**Figure 6:**
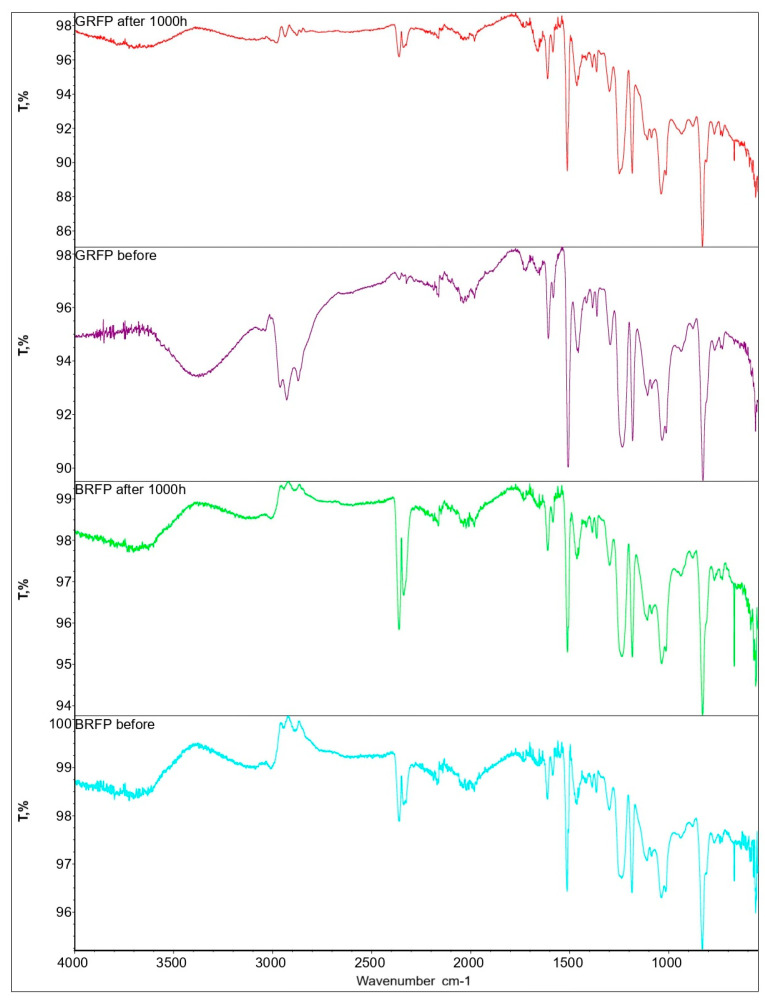
FTIR spectra of GFRPs and BFRPs before and after 1000 h irradiation (λ = 340 nm), showing changes in C–H (2800–3000 cm^−1^), C=O (~1730 cm^−1^), and O–H (~3400 cm^−1^) regions.

Fourier-transform infrared (FTIR) spectroscopy was employed to monitor chemical changes in GFRP and BFRP samples after UV exposure (λ = 340 nm, 1000 h). Figure 6 presents comparative FTIR spectra of before exposure and after radiation exposure of GFRPs (samples filled with glass fiber) and BFRPs (samples filled with basalt fiber).

The key spectral regions analyzed include 2800–3000 cm^−1^ (C–H stretching vibrations), ~1730 cm^−1^ (carbonyl C=O stretching), and ~3400 cm^−1^ (O–H stretching). The spectra show that after UV irradiation, GFRP samples exhibit an increase in peak intensity in these regions, indicating the development of photo-oxidative degradation processes.

In contrast, the BFRP spectrum only shows minimal changes, indicating higher resistance to UV-induced degradation. According to the literature, basalt fiber suppresses the formation of carbonyl and hydroperoxide groups 70–85% more effectively than glass fiber [30], due to its mineral composition (e.g., FeO, TiO_2_), which enables UV absorption and radical neutralization [31].

Thus, the FTIR findings are consistent with previously recorded changes in thickness and mass and confirm that the dimensional instability of GFRPs is linked to photo-oxidative matrix degradation, while the stability of BFRP is due to its inertness to UV-destructive processes. Nevertheless, considering the limitations of measurement accuracy, these conclusions should be regarded as preliminary and require further confirmation through thermal (DSC, TGA) and morphological (SEM) analyses.

To estimate the penetration depth of UV-induced effects on laminated polymer composite materials (PCMs), a calculation was performed based on the relationship between material mass loss, density, and the irradiated surface area. It is assumed that mass loss occurs primarily due to the removal of the surface layer as a result of photo- and thermal degradation, and that this process predominantly affects one side of the sample.

(1) $$h=\frac{\Delta m}{\rho *A},$$

where Δ*m* is the mass loss (g), *ρ* is the material density (g/cm^3^), *A* is the irradiated surface area (cm^2^), and ℎ is the thickness of the degraded layer (cm).

This approach is justified under the assumption of uniform material removal from the surface without internal failure and is widely used in studies of climatic aging and the photodegradation of polymers [32].

The application of this formula aligns with methodological guidelines for surface degradation analysis as outlined in international standard ISO 4892-1:2016 [33] and in research on the durability of polymer coatings [34]. Similar methods for calculating degradation depth have been employed in a number of experimental studies focused on evaluating the longevity of epoxy-based materials under UV exposure [35].

To quantitatively assess the degree of surface degradation of GFRPs and BFRPs after 2000 h of UV irradiation, the degradation depth was calculated based on the recorded mass loss of the samples.

This allows for an objective comparison of the materials’ resistance to UV-induced damage and provides a reliable indicator of structural stability under environmental stressors.

For Glass Fiber-Reinforced Plastic (GFRP),

*Δm* = 0.02; *ρ* = 1.607 g/cm^3^; *A* = 22 cm^2^

$$h=\frac{0.0200}{1.607*22}=5.66\;µm$$

For Basalt Fiber-Reinforced Plastic (BFRP),

*Δm* = 0.015; *ρ* = 1.369 g/cm^3^; *A* = 22 cm^2^

$$h=\frac{0.0151}{1.369*22}=5.01\;µm$$

Thus, after 2000 h of UV exposure, the degraded layer depth was determined to be 5.66 µm for GFRPs and 5.01 µm for BFRPs. These results are consistent with findings from similar studies on the surface degradation of polymeric materials.

During the experiment, the surfaces of the laminated plastics were analyzed using a contact profilometer Mitutoyo SJ-201, designed for the high-precision measurement of surface microgeometry parameters. This device enables the acquisition of both quantitative roughness metrics (e.g., Ra, Rz) and surface profiles in the form of profilograms, which is particularly valuable for assessing the effects of UV aging.

To ensure the reliability of the profilometric analysis, measurements were conducted on at least three specimens of each material type, and in three distinct surface zones per specimen. The obtained data were subjected to statistical processing, with the average surface roughness parameter (Ra) calculated for each set.

**Figure 7:**
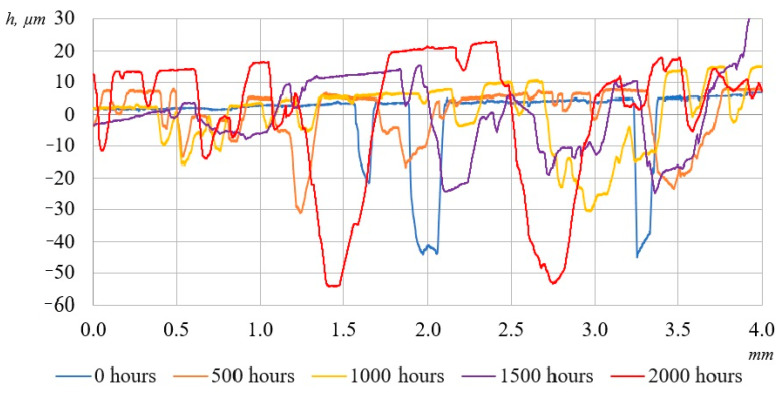
Surface profilograms of BFRP after UV exposure for 0, 500, 1000, 1500, and 2000 h.

**Figure 8:**
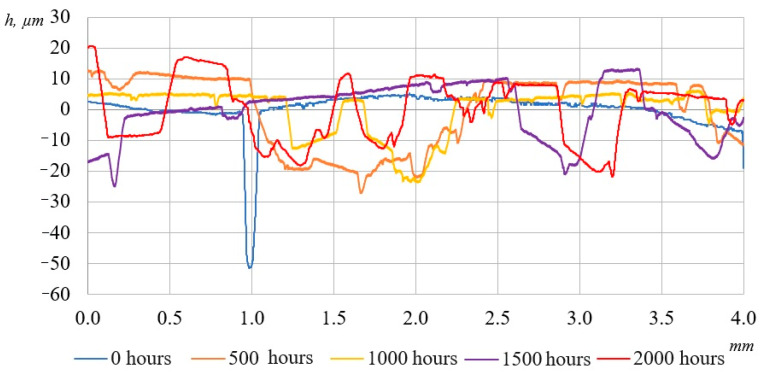
Surface profilograms of GFRPs after UV exposure for 0, 500, 1000, 1500, and 2000 h.

Figure 7 and Figure 8 show the surface representative profilograms of the composites, where significant changes can be observed. As seen in the figures, after 2000 h of UV exposure, the samples underwent noticeable alterations compared to their initial states. The surface became distinctly rougher. These changes are attributed to the UV-induced degradation of the surface layer, leading to the formation and enlargement of pores in the epoxy matrix.

Numerical values of changes in average surface roughness (Ra) of the samples are shown in Table 4.

As shown in Table 4, the average surface roughness (Ra) values steadily increase with prolonged UV exposure time, with distinct behavior observed between BFRP and GFRP samples.

In the early stages of UV exposure (500 h), BFRP samples exhibit a slight decrease in surface roughness (−4.96%), likely due to the additional densification of the resin under the thermal effect of the UV lamps. However, after 1000 and 1500 h, a sharp increase in BFRP roughness is observed (+10.4% and +46.7%, respectively), attributed to the onset of matrix degradation, microcrack formation, and the partial exposure of the fiber reinforcement. By 2000 h, the roughness of BFRP increased by +94%, indicating the significant progression of photodegradation and the development of a more pronounced surface microrelief.

In contrast, GFRP samples demonstrate a consistent increase in surface roughness even in the early stages of irradiation (+15.35% after 500 h), which continues through 1500 h (+138.6%). After 2000 h, the roughness of GFRPs increased by 241.2% compared to the initial state, indicating significantly more intense degradation of the epoxy matrix. This points to the lower resistance of GFRPs to prolonged UV exposure, characterized by the early onset of surface deterioration and reduction in protective properties.

Overall, BFRP exhibits greater stability during the initial stages of UV exposure, but a sharp rise in roughness occurs toward the end of testing. GFRPs, on the other hand, show earlier and more intense surface degradation, highlighting their lower resistance to UV radiation. These results suggest that while BFRPs are more resilient to short-term UV effects, they become increasingly vulnerable under prolonged exposure. In contrast, GFRPs display continuous surface deterioration from the very beginning of the exposure period.

Changes in Flexural Strength and Modulus of Elasticity After 2000 Hours of UV Exposure

To assess the elastic and strength characteristics of the composites, a series of flexural tests were conducted in accordance with GOST R 56810-2015 [36]. The tests were performed using a Zwick Roell Z600 universal testing machine (Zwick, Ulm, Germany), model VRS-F0600TN.R09, serial number: 160088-2008, at the Shared Research Facilities of the Federal Research Centre, “The Yakut Scientific Centre of the Siberian Branch of the Russian Academy of Sciences”.

To eliminate the plasticizing effect of moisture, all composite specimens were pre-dried at 60 °C for 72 h prior to mechanical testing. In accordance with GOST, the specimens were prepared with dimensions of 20 × 110 ± 0.2 mm, with five samples tested for each material type.

During testing, the samples were placed on supports with a span of 80 mm and were loaded at a constant rate of 2 mm/min until failure. The radius of the loading punch was 5 mm.

**Figure 9:**
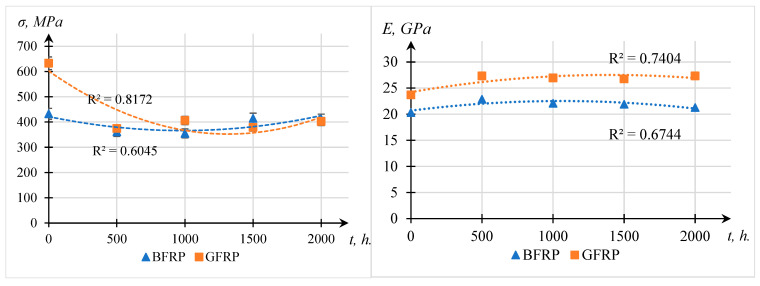
Dependence of flexural strength (σ) and modulus of elasticity (E) on UV exposure time (t, h) for composite plastics.

The results of the flexural tests for the composite plastics are presented in Figure 9, where: *Ei*—modulus of elasticity, GPa; *σi*—flexural strength, MPa

These parameters provide a comprehensive measure of the structural response of the materials after prolonged UV exposure.

Figure 9 shows the nonlinear dependence of flexural strength and elastic modulus of composite plastics on UV exposure time. The initial decrease in strength (up to 1000 h) is attributed to the degradation of the polymer matrix surface layer: under UV radiation, photochemical bond scission, microcrack formation, and the weakening of fiber–matrix interfacial adhesion occur. Between 1000 and 1500 h, a partial recovery in strength is observed, which may be related to the post-curing of the epoxy resin under residual heat and UV exposure, as well as structural densification due to the relaxation of internal stresses.

These processes are consistent with the FTIR spectroscopy results (Figure 6), which show a decrease in the intensity of C–H absorption bands and the emergence of carbonyl peaks, confirming photochemical changes in the matrix. However, further exposure beyond 1500 h leads to the accumulation of degradation damage and a sustained decline in mechanical properties.

Similarly, the early-stage increase in elastic modulus is explained by post-curing effects that enhance the stiffness of the matrix. The decline after 1500 h reflects the predominance of degradation mechanisms, such as microcrack formation and weakened fiber–matrix adhesion. These trends indicate the existence of a competitive interplay between structural reinforcement and photodegradation processes during prolonged UV aging.

The obtained test results indicate that after 500 h of UV exposure, both GFRP and BFRP samples exhibited a reduction in flexural strength (σи), with the maximum strength loss reaching 41% for GFRPs. This may be attributed to interlaminar shear failure in the composite, which is typical for systems with a weak matrix or insufficient adhesion between the matrix and the reinforcing fiber [37].

To assess the impact of 2000 h of UV irradiation on the elastic-strength properties of the composites, a generalized indicator was introduced. This was the relative retention coefficient, defined as follows:

(2) $$k_{R}=R_{t}/R_{0},\;$$

where *R*_*t*_ is the flexural strength value obtained after UV chamber exposure, and *R*_0_ is the corresponding strength of the unexposed (initial) material.

Table 5 presents the overall results of elastic-strength property testing for each composite type.

Based on the analysis of mechanical testing by the three-point bending method, it was established that UV exposure leads to a reduction in strength for both BFRP and GFRP composites, although the degradation patterns differ significantly.

BFRPs exhibit considerably higher resistance to photodegradation: after 500 h of UV exposure, their flexural strength decreases only to 83% of the initial value and remains at 95% after 2000 h. In contrast, GFRPs degrade much more intensively, with strength dropping to 59% after just 500 h, and reaching only 64% of the original value after 2000 h.

The marked reduction in GFRP strength may be attributed to the surface layer degradation and delamination of one of the outer reinforcement layers, as shown by Goel et al. [38]. A likely cause of delamination is localized failure of interfacial bonding between the matrix and the fibers, induced by elevated UV exposure [18].

One factor that may intensify photodegradation in polymer composites under UV radiation is the interference effect, which arises from the alternating layers with differing optical properties. This alternation enhances radiation reflection at certain wavelengths, resulting in uneven degradation particularly at the matrix–fiber interfacial zone.

The influence of surface structure and optical characteristics is corroborated by field test results reported by Nizina T.A. et al. [39], who demonstrated that the UV resistance of epoxy composites significantly depends on the color and saturation of the coating. Over a 10-month outdoor exposure, black and brown composites with high initial color saturation exhibited minimal loss of decorative appearance and retained a more stable structure compared to transparent and grey counterparts. This behavior is attributed to the high UV-absorbing capacity of dark pigments, effectively limiting the penetration depth of radiation into the subsurface matrix layers.

Similar conclusions were reached in a study by Colonna et al., which investigated the photostabilization of polypropylene films modified with different pigments. It was shown that the incorporation of dark pigments (black, red) significantly reduces photodegradation intensity due to their ability to absorb UV radiation and dissipate energy. This provides the long-term protection of both the optical and mechanical properties of the polymer matrix during outdoor service [40].

In conclusion, the structure and composition of the composite’s surface layer are critical to its aging resistance. For the BFRP composite, which naturally exhibits dark coloration, the observed lower degree of degradation is explained by a combination of the following factors: efficient absorption of ultraviolet radiation by the dark surface layer; reduced radiation penetration depth to the interfacial boundary; delayed photo-oxidation of the polymer matrix in the near-surface zone.

These combined effects confer increased UV resistance to BFRP composites and underscore the importance of carefully selecting the surface layer when designing long-lasting composite materials.

Comparative analysis of degradation in GFRPs and BFRPs under UV chamber and natural outdoor exposure

To validate the reliability of the laboratory model of accelerated aging, comparative analysis was conducted between the results of UV exposure in a climatic chamber and natural weathering in Yakutsk—one of the regions with the most extreme climatic conditions in the world. Laboratory UV aging was carried out for 2000 h at a constant intensity of 7.88 W/m^2^. To calculate the equivalent exposure time under Yakutsk conditions, climate data for the years 2022–2024, provided by the Federal State Budgetary Institution “Yakutsk Department for Hydrometeorology and Environmental Monitoring,” were used (Table 6).

The average value of the UV component of solar radiation was *D*_UVavg_ = 85.76 kWh/m^2^/year.

The laboratory chamber, with an intensity of 7.88 W/m^2^, provided an annual exposure dose of *D*_lab_ = 69.05 kWh/m^2^, resulting in the ratio of laboratory to natural UV dose being

$$K_{D}=\frac{69.05}{85.76}=0.81$$

To account for thermal acceleration, the Arrhenius equation was used [41,42]:

(3) $${K_{T}=e}^{\frac{Ea}{R}(\frac{1}{Tnat}-\;\frac{1}{Tlab})}$$

where *E_a_* is activation energy, typically expressed in kJ/mol or J/mol, R is universal gas constant, 8.314 J/(mol·K), *T*_nat_ is temperature under natural exposure conditions in Kelvin (K), and *T*_lab_ is laboratory temperature in Kelvin (K).

(4) $$K_{T}\approx \;e^{\frac{50000}{8.314}\left(\frac{1}{266\;}-\frac{1}{298}\right)}\approx \;e^{2.53}\approx 12.6$$

Thus, the total acceleration factor is

K_total_ = K_D_ × K_T_ = 0.81 × 12.6 ≈ 10.14

Indeed, 2000 h of exposure correspond to 0.228 years (2000/8760). Then, the equivalent exposure time under Yakutsk climate conditions is

t_eq_ = 0.228 × 10.14 ≈ 2.31 years

Therefore, 2000 h of laboratory UV exposure in the test chamber correspond to approximately 2.3 years of natural aging under the extreme climatic conditions of Yakutsk.

As part of the work under the State Assignment FWRS-2024-0058, analysis was conducted on the changes in the physical and mechanical properties of GFRP and BFRP samples after natural exposure in Yakutsk for a period of 36 months. Table 7 presents the results of three-point bending tests on the samples after 12, 24, and 36 months of outdoor exposure in Yakutsk.

The comparison of mechanical test results after laboratory UV exposure and natural exposure under the extremely cold climate conditions of Yakutsk confirms the validity of the calculation and makes it possible to identify the specific aging behavior of GFRPs and BFRPs under different environmental influences.

Glass Fiber-Reinforced Plastics (GFRPs):

Under laboratory conditions, the flexural strength of GFRPs dropped by 41% after just 500 h of UV exposure (retention coefficient *k*_*R*_ = 0.59). By contrast, under natural exposure conditions, the strength decreased by only 9% after 12 months (*k*_*R*_ = 0.91) and by 29% after 24 months (*k*_*R*_ = 0.71). After 36 months, a partial recovery of strength is observed, reaching a retention coefficient of *k*_*R*_ = 0.89, which may be attributed to the post-curing of the polymer matrix under prolonged exposure to temperature and humidity, as well as to the redistribution of internal stresses within the material. These results indicate that degradation proceeds 1.5 to 2 times faster in laboratory tests, largely due to the continuous and high-intensity UV irradiation in the chamber compared to the fluctuating and intermittent exposure in the natural environment (e.g., daily and seasonal variations in temperature, humidity, cloud cover, and snow presence).

Basalt Fiber-Reinforced Plastics (BFRP):

While laboratory UV irradiation also caused some degradation in BFRPs, the effect was significantly less pronounced: after 500 h, *k*_*R*_ = 0.83, and after 2000 h, the material retained 95% of its original strength. Under natural exposure, the flexural strength of BFRP remained virtually unchanged after 12 months (*k*_*R*_ = 1.01) and was still 82% of the initial value after 24 months (*k*_*R*_ = 0.82). After 36 months, the ultimate strength, on the contrary, exceeds the initial value by 25% (*k*_*R*_ = 1.25). This may be due to the effect of long-term post-curing of the epoxy matrix, structural densification, and the stabilization of interfacial interactions. These results highlight the high resistance of basalt fiber composites to aggressive climatic conditions and their ability to undergo structural self-strengthening during prolonged service.

Basalt fiber composites, due to the naturally dark color of the fiber, exhibit the ability to shield UV radiation in the surface layers, thereby limiting the penetration depth of destructive energy. In contrast, in glass fiber composites, the lighter color of the reinforcing fibers allows photodegradation processes to propagate more rapidly into the material’s depth.

**Figure 10:**
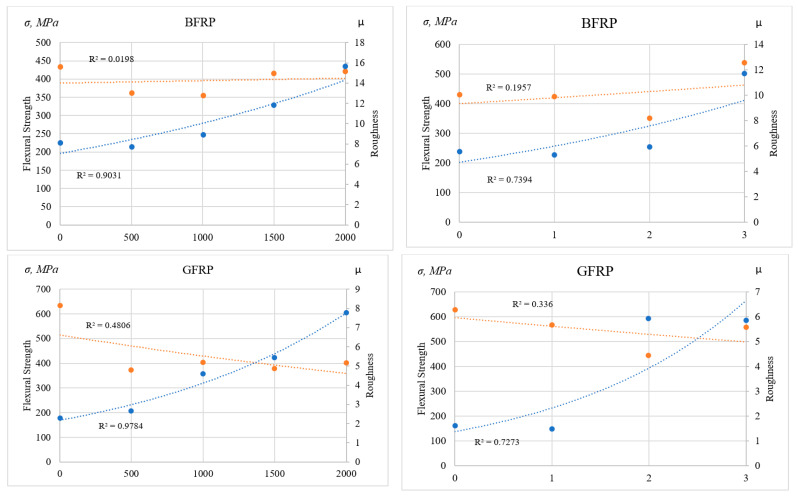
Dependence of flexural strength on average surface roughness (Ra) after 2000 h of UV exposure (**left**) and after 36 months of natural weathering (**right**).

Both under laboratory accelerated aging and during natural exposure, an initial decrease in the elastic modulus is observed, caused by the degradation of the composite’s surface layer, as confirmed by profilometric measurements. Figure 10 shows the relationship between flexural strength and average surface roughness (Ra) after 2000 h of UV exposure (left) and after 36 months of natural exposure (right).

The analysis of the results from both natural weathering and accelerated UV aging revealed fundamentally different behaviors of basalt fiber-reinforced plastics (BFRPs) and glass fiber-reinforced plastics (GFRPs), driven by the nature of their structural transformations under external influences. During natural exposure, BFRPs showed a moderate strength reduction (~18%) and consistently low roughness (up to 6 µm) over the first two years, but exhibited an unexpected increase in strength to 535.8 MPa in the third year, accompanied by a sharp rise in roughness to 11.7 µm. This may indicate delayed thermo- or climate-induced recrystallization or crosslinking processes. Under UV aging, BFRP also exhibited an initial decrease in strength (down to 355 MPa), followed by partial recovery, likely due to photostimulated structural reorganization, despite intense surface degradation (roughness up to 15.7 µm).

In contrast, GFRPs demonstrated a steady decrease in strength under both exposure modes (down to ~444 MPa for natural weathering and ~402 MPa in the UV chamber), along with a significant increase in surface roughness (up to 6–7.8 µm), suggesting dominant photo-destructive mechanisms without signs of structural stabilization. However, a partial recovery of strength to 556.9 MPa was observed in the third year of natural exposure, possibly linked to stress relaxation or microdefect redistribution.

A particularly noteworthy finding is the correlation between water retention and surface roughness. Increased roughness (especially above 10 µm) promotes the retention of bound water in micro-roughness zones, particularly under high humidity and low temperature. Bound water is known to act as a plasticizer, reducing matrix brittleness, stabilizing volume, and slowing down degradation of the matrix [43,44]. This effect is described in studies [45,46], which show that water adsorbed on rough surfaces or in micropores delays aging processes and enhances the resistance of polymer materials to environmental degradation.

Under UV aging, where light exposure is accompanied by elevated temperature, the evaporation of free moisture is intense. However, in micropores and rough depressions, water evaporates more slowly and remains in a bound state, enabling localized thermal regulation and slowing degradation. This explains why BFRPs, with its higher surface roughness, demonstrate greater resistance to photodegradation and retain mechanical strength under long-term exposure. It also accounts for the absence of significant thickness changes at early exposure stages: moisture retained in bound form prevents shrinkage and structural deformation that typically occur in smoother materials. In contrast, GFRPs, with their initially smoother surface and lower microporosity, lack such water retention capability and lose moisture more rapidly, especially under cyclic high temperatures in the UV chamber, leading to shrinkage, delamination, and accelerated degradation.

Thus, laboratory testing in a specially designed UV chamber enables reliable modeling of aging processes in polymer composites. However, the interpretation of the results requires the consideration of differences in exposure intensity and duration between laboratory and natural environments. The direct comparison of the data confirms the superior durability of BFRPs over GFRPs in both accelerated and natural climatic aging conditions.

## 5. Conclusions

The research conducted confirmed that the durability of polymer composite materials under UV aging is determined by the combined effect of the optical properties of the fibers, the characteristics of the polymer matrix, and the surface microgeometry.

During accelerated aging tests, it was established that GFRP composites exhibit high sensitivity to UV exposure, manifested by a reduction in strength, an increase in microdefects, and the development of degradation processes within the polymer phase. In contrast, BFRP composites demonstrate significantly higher resistance due to the shielding properties of basalt fibers, including their ability to absorb UV radiation and inhibit the progression of photo-oxidative reactions.

Key degradation mechanisms include the destruction of interfacial adhesion, formation of microcracks, the oxidation of the matrix, and local stresses induced by moisture loss. At the same time, processes such as post-curing, moisture retention, and surface stabilization may slow down deterioration and partially compensate for strength loss.

The developed model of laboratory UV aging has proven effective for predicting material behavior under real conditions and can be used for accelerated assessment of the climate resistance of composite structures.

## Figures and Tables

**Table 1:** Composition and properties of components used in investigated FRP materials.

FRP Component	Parameter	Value
Epoxy resin CYD-128	Color (Fe-Co scale)	1
	Epoxy group content, wt.%	21.4
	Chloride ion content, wt.%	0.0009
	Saponifiable chlorine content, wt.%	0.4
	Volatile matter content, wt.%	0.3
	Dynamic viscosity at 25 °C, Pa·s	16
	Hydroxyl group content, wt.%	1.5
Curing agent iso-MTHPA	Main substance content, wt.%	99.2
	Viscosity (VZ-4 viscometer) at 20 °C, s	28
	Gel time at 150 °C, h	8
	Acid number, wt.%	2
Accelerator UP-606/2	Main substance content, wt.%	98.4
	Titrated nitrogen content, wt.%	14
	Density at 25 °C, kg/m^3^	10
Glass roving fabric TR-560-30A	Surface density, g/m^2^	720 ± 50
	Tensile strength (warp), N	3500
	Tensile strength (weft), N	3500
	Warp yarn density, threads/cm	30 ± 1
	Weft yarn density, threads/cm	30 ± 1
	Weave type	plain weave
Basalt fabric BT-11	Surface density, g/m^2^	351 ± 20
	Tensile strength (warp), N	2610
	Tensile strength (weft), N	2120
	Warp yarn density, threads/cm	18
	Weft yarn density, threads/cm	8
	Weave type	twill 5/3

**Table 2:** Composition and layer structure of composite samples.

Material Name	Sample Code	Number of Fabric Layers, pcs.	Thickness h, mm
Glass fiber-reinforced plastic	GFRP	14	5.15 ± 0.1
Basalt fiber-reinforced plastic	BFRP	18	5.28 ± 0.1

**Table 3:** Thickness values of laminated composites subjected to UV radiation.

Material	0 h	500 h	1000 h	1500 h	2000 h
GFRP	5.27 ± 0.01	5.27 ± 0.02	5.26 ± 0.01	5.27 ± 0.02	5.21 ± 0.03
BFRP	5.40 ± 0.02	5.36 ± 0.02	5.38 ± 0.02	5.36 ± 0.01	5.38 ± 0.02

**Table 4:** Average surface roughness (Ra) values of laminated composites after UV exposure.

Material	0 h, µm	500 h, µm	1000 h, µm	1500 h, µm	2000 h, µm
GFRP	2.28	2.63	4.61	5.44	7.78
BFRP	8.07	7.67	8.91	11.84	15.66

**Table 5:** Results of three-point bending tests of samples after UV exposure for 0, 500, 1000, 1500, and 2000 h.

Material	UV Exposure Time	Flexural Strength			Retention Coefficient
	t, h	σ, MPa	Std. Dev.	Error	kR
GFRP	0	632.73	31.22	13.96	1.00
	500	374.5	22.93	10.25	0.59
	1000	406.1	11.58	5.18	0.64
	1500	378.87	29.35	13.13	0.60
	2000	402.05	24.5	10.96	0.64
BFRP	0	432.54	24.14	10.79	1.00
	500	361.05	28.82	12.89	0.83
	1000	355.09	23.81	10.65	0.82
	1500	414.57	62.63	28.01	0.96
	2000	410.83	74.92	33.51	0.95

**Table 6:** Characteristics of the extreme cold climate in Yakutsk.

Climate Characteristic	2022	2023	2024
Average annual air temperature, °C	−6.9	−7.8	−6.94
Average annual relative humidity, %	67.7	68.1	62.09
Absolute maximum air temperature, °C	35.0	35.5	32.2
Absolute minimum air temperature, °C	−48.7	−51.9	−47.2
Annual total precipitation, mm	265	268	173.5
Annual total solar radiation, MJ/m^2^	301.7	332.8	291.7
Average annual wind speed, m/s	1.5	1.6	1.6

**Table 7:** Results of three-point bending tests after exposure in Yakutsk for 12, 24 and 36 months.

Material	Indicator	Initial (0)	After 12 Months (K-12)	k_R_(12)	After 24 Months (K-24)	k_R_(24)	After 36 Months (K-36)	k_R_(36)
GFRP	σ, MPa	625.93 ± 13.28	568.41 ± 18.64	0.91	444.38 ± 12.12	0.71	556.92 ± 35.0	0.89
	E, GPa	23.92 ± 0.28	22.91 ± 0.36	0.96	18.04 ± 0.30	0.75	24.86 ± 0.66	1.04
BFRP	σ, MPa	428.41 ± 13.57	432.16 ± 10.60	1.01	351.97 ± 8.8	0.82	535.79 ± 10.82	1.25
	E, GPa	20.31 ± 0.23	22.64 ± 0.20	1.11	17.35 ± 0.17	0.85	42.67 ± 2.04	2.10

## Data Availability

The original contributions presented in the study are included in the article, further inquiries can be directed to the corresponding author.

## References

[1] Ali S., Liu J., Zheng H., Zhang Y., Chen Y. (2024). Durability of Basalt and Glass Fiber Composites Under Extreme Environments. Polym. Compos..

[2] Du H., Xian G., Tian J., Ma Z., Li C., Xin M., Zhang Y. (2025). Effect of fiber surface treatment with silane coupling agents and carbon nanotubes on mechanical properties of carbon fiber reinforced polyamide 6 composites. Polym. Compos..

[3] Lu Z., Jiang M., Pan Y., Xian G., Yang M. (2022). Durability of basalt fibers, glass fibers, and their reinforced polymer composites in artificial seawater. Polym. Compos..

[4] Mariam M., Belouettar S., Ayadi Z. (2019). Influence of hydrothermal ageing on the mechanical properties of an adhesively bonded joint with different adherends. Compos. Part B Eng..

[5] Reis J.M.L., da Costa Mattos H.S., Ferreira A.J. (2017). Influence of ageing in the failure pressure of a GFRP pipe used in oil industry. Eng. Fail. Anal..

[6] Zepp R.G., Acrey B., Davis M.J.B., Andrady A.L., Locklin J., Arnold R., Okungbowa O., Commodore A. (2023). Weathering effects on degradation of low-density polyethylene-nanosilica composite with added pro-oxidant. J. Polym. Environ..

[7] Andreyeva N.P., Pavlov M.R., Nikolayev E.V., Kurnosov A.O. (2019). Study of the influence of atmospheric factors on the properties of polymer structural fiberglass on a cyanoester base in natural conditions of cold, moderate and tropical climates. Proc. VIAM..

[8] Korkmaz Y., Gültekin K. (2022). Effect of UV irradiation on epoxy adhesives and adhesively bonded joints reinforced with BN and B4C nanoparticles. Polym. Degrad. Stab..

[9] Satdinov R.A., Veshkin E.A., Postnov V.I. (2020). Assessment of the impact of climatic factors on the performance properties of fiberglass grade VPS-42P/T-64. Proc. VIAM..

[10] Vasilyeva E.D., Vasilyeva A.A., Kychkin A.K. (2022). On the methods of studying moisture effects on polymer composite materials. Mater. Sci. Energy.

[11] Startsev V.O., Valevin E.O., Gulyaev A.I. (2020). The influence of surface aging of polymer composites on their mechanical properties. Proc. VIAM..

[12] Kablov E.N., Startsev V.O. (2021). Effect of internal stresses on aging of polymer composite materials. A review. Mech. Compos. Mater..

[13] Lukachevskaya I.G., Kychkin A.K., Gavrilieva A.A., Kychkin A.A., Lebedev M.P. (2021). Assessment of the initial stage of climatic ageing of basalt and glass-reinforced plastics in extremely cold climates. Nat. Resour. Arct. Subarct..

[14] Starkova O., Aniskevich K., Sevcenko J. (2021). Long-term moisture absorption and durability of FRP pultruded rebars. Mater. Today Proc..

[15] Li C., Xian G., Li H. (2019). Combined effects of temperature, hydraulic pressure and salty concentration on the water uptake and mechanical properties of a carbon/glass fibers hybrid rod in salty solutions. Polym. Test..

[16] Gavrilieva A.A., Startsev O.V., Lebedev M.P., Krotov A.S., Kychkin A.K., Lukachevskaya I.G. (2024). Size Effects in Climatic Aging of Epoxy Basalt Fiber Reinforcement Bar. Polymers.

[17] Lu T., Solis-Ramos E., Yi Y., Kumosa M. (2018). UV degradation model for polymers and polymer matrix composites. Polym. Degrad. Stab..

[18] Shi Z., Zhang H., Zhang Y., Wang Y., Zhang J. (2022). Analysis of the Mechanical Properties and Damage Mechanism of Carbon Fiber/Epoxy Composites under UV Aging. Materials.

[19] Wu C., Meng B.C., Tam L.-H., He L. (2022). Yellowing mechanisms of epoxy and vinyl ester resins under thermal, UV and natural aging conditions and protection methods. Polym. Test..

[20] Khotbehsara M.M., Farahani H., Karimpour A., Alipour M. (2020). Effects of Ultraviolet Solar Radiation on the Properties of Particulate-Filled Epoxy-Based Polymer Coating. Polym. Degrad. Stab..

[21] Hashim U.R., Jumahat A., Jawaid M., Dungani R., Alamery S. (2020). Effects of Accelerated Weathering on Degradation Behavior of Basalt Fiber Reinforced Polymer Nanocomposites. Polymers.

[22] Kazi S.N., Dharan C.K.H., Nizamuddin S. (2017). Effect of UV Radiation on the Mechanical Properties of Basalt Fiber Reinforced Epoxy Composites. Mater. Today Proc..

[23] (2023). Standard Practice for Operating Fluorescent Ultraviolet (UV) Lamp Apparatus for Exposure of Materials.

[24] Tanks J., Naito K. (2022). UV durability assessment of a thermoplastic epoxy-based hybrid composite rod for structural reinforcement and retrofitting. J. Build. Eng..

[25] Kurs M.G., Nikolaev E.V., Abramov D.V. (2019). Natural and accelerated tests of metal and non-metal materials: Key factors and test rigs. Aviation Mater. Technol..

[26] Chin J.W. (2007). Durability of Composites for Civil Structural Applications.

[27] Nikolaev E.V., Barbotko S.L., Andreeva N.P., Pavlov M.R., Grashchenkov D.V. (2016). Comprehensive study of the effects of climatic and operational factors on a new generation of epoxy matrix composites. Proc. VIAM..

[28] Pavlov M.R., Nikolaev E.V., Andreeva N.P., Barbotko S.L. (2016). On the methodology for evaluating the resistance of polymer materials to solar radiation. Proc. VIAM..

[29] (2016). Plastics. Determination of Thickness of Plastic Films and Sheeting by Mechanical Scanning (ISO 4593:1993, IDT).

[30] Wan Y., Yan H., Xie L., Li J., Wang J. (2015). UV Degradation Resistance of Basalt Fiber Reinforced Polymer Composites Compared to Glass Fiber Counterparts. Compos. B Eng..

[31] Petousis M., Vidakis N., Gkartzonikas A., Mountakis N., Grammatikos S.A. (2022). A Study on the UV Aging of Basalt and Glass Fiber Reinforced Epoxy Composites.

[32] Gugumus F. (2001). Photodegradation and photooxidation of thermoplastics. Polym. Test..

[33] (2016). Plastics—Methods of Exposure to Laboratory Light Sources—Part 1: General Guidance.

[34] Trubachev S.G., Matveev A.V. (2012). Fundamentals of degradation modeling for polymer coatings. Surf. Phenom. Physicochem. Mech..

[35] Krzeminski M., Puchalski M., Gozdecki C. (2015). Surface degradation of polymer materials under UV radiation. Polym. Degrad. Stab..

[36] (2016). Polymer Composites. Method of Testing Flat Specimens by Bendingstate.

[37] Richardson M., Babaevsky P.G. (1980). Industrial Polymer Composite Materials.

[38] Goel A., Chawla K.K., Vaidya U.K., Koopman M., Dean D.R. (2008). Effect of UV exposure on the microstructure and mechanical properties of long fiber thermoplastic (LFT) composites. J. Mater. Sci..

[39] Nizina T.A., Chernov A.N., Nizin D.R., Popova A.I. (2016). Influence of color on the colorimetric stability of epoxy composites under natural weathering. Vestn. MGSU..

[40] Colonna G.M., Paci M., Chiellini E. (2006). Photostabilization of polypropylene films by pigment and additive combinations. J. Appl. Polym. Sci..

[41] Bernstein L.S., Kotlyarov V.I. (2018). Kinetics of Thermo-Oxidative Aging of Polymeric Materials.

[42] Gusev P.M., Koroleva T.A. (2020). Modeling of Polymer Aging Processes under Various Temperature Conditions.

[43] Caurie M. (2011). Bound water: Its definition, estimation and characteristics. Int. J. Food Sci. Technol..

[44] Abdelmola F., Carlsson L.A. (2019). State of water in void–free and void–containing epoxy specimens. J. Reinf. Plast. Compos..

[45] Zafeiropoulos N., Williams D., Baillie C., Matthews F. (2002). Engineering and characterisation of the interface in flax fibre/polypropylene composite materials. Part I. Development and investigation of surface treatments. Compos. Part A Appl. Sci. Manuf..

[46] Apicella A., Migliaresi C., Nicodemo L., Nicolais L., Iaccarino L., Roccotelli S. (1982). Water sorption and mechanical properties of a glass-reinforced polyester resin. Composites.

